# A Case of Metastatic Lung Adenocarcinoma, With Metastasis to the Brain, Spleen, Liver, and Bone, Mimicking Sarcoidosis

**DOI:** 10.7759/cureus.95646

**Published:** 2025-10-29

**Authors:** Dalal A Obaid, Saara Ismail, Meera Al Shirawi, Jawad ElHaout, Priyadarshini Chaudhary, Katie Sweeting

**Affiliations:** 1 Medicine, College of Medicine, Mohammed Bin Rashid University of Medicine and Health Sciences, Dubai, ARE; 2 Internal Medicine, College of Medicine, Mohammed Bin Rashid University of Medicine and Health Sciences, Dubai, ARE; 3 Internal Medicine, NMC Royal Hospital, Dubai, ARE; 4 Radiology, Mediclinic City Hospital, Dubai, ARE; 5 Oncology, Mediclinic City Hospital, Dubai, ARE

**Keywords:** anchoring bias, bilateral hilar lymphadenopathy, brain metastases, diagnostic delay, fdg pet ct, lung adenocarcinoma, neurosarcoidosis, ros1 rearrangement, sarcoidosis mimicry, tissue biopsy

## Abstract

Lung adenocarcinoma can radiologically and clinically mimic granulomatous disease, leading to diagnostic delays. Bilateral hilar and mediastinal lymphadenopathy, perilymphatic nodules, and partial corticosteroid responsiveness may anchor clinicians to a diagnosis of sarcoidosis. This case highlights the importance of maintaining a broad differential and pursuing a timely, tissue-based diagnosis when features evolve atypically.

We report a previously healthy 49-year-old male, lifelong non-smoker, who presented with three months of progressive multisystem symptoms, including exertional dyspnea, nocturnal cough, constitutional complaints, neuropathic pain, visual auras, and intermittent paresthesia. Imaging revealed diffuse thoracic lymphadenopathy, pulmonary fibrosis, splenic and adrenal nodules, and multifocal enhancing brain lesions, while positron emission tomography (PET)-computed tomography (CT) demonstrated widespread fluorodeoxyglucose (FDG)-avid disease. Although sarcoidosis and infective granulomatous conditions were initially considered, a cervical lymph-node biopsy confirmed metastatic lung adenocarcinoma (stage IV). This case illustrates how radiologic mimicry and anchoring bias can delay the recognition of malignancy, emphasizes the non-specific nature of FDG avidity, and underscores the decisive role of histological confirmation. Early consideration of malignancy in “sarcoid-like” presentations, especially when systemic features or incomplete steroid response arise, is essential to ensure timely oncologic therapy and integration of supportive care.

## Introduction

Lung adenocarcinoma, a prevalent subtype of non-small cell lung cancer (NSCLC), holds the distinction of being the most common primary lung cancer in the United States [1]. Globally, lung cancer accounts for 11.6% of newly diagnosed cancers [2]. Despite its strong correlation with tobacco use, lung adenocarcinoma is frequently diagnosed in non-smokers [1]. Vague and nonspecific symptoms may be misleading and point toward other diagnoses, ultimately causing a delay. As a result, only a small proportion of NSCLC patients are diagnosed at a stage where treatment is curative [3].

Risk factors for lung cancer include primary or secondary tobacco exposure, a positive family history of lung cancer, and environmental factors, such as occupational exposure to asbestos, silica, and heavy metals that can induce genetic mutations, particularly in the* p53* gene, which normally prevents uncontrolled cell division [1]. Adenocarcinoma predominantly arises in the peripheral regions of the lung, unlike other NSCLC subtypes that often present as central lesions. Symptoms include dyspnea, cough, and, in more advanced stages, pleural effusion. Delayed diagnosis may also reveal systemic manifestations, like unexplained weight loss or cachexia, due to metastasis [4].

Initial diagnostic imaging includes chest X-ray, which may show a ground-glass solid nodule or opacity [5], and hilar and mediastinal adenopathy, which is nonspecific and may mimic granulomatous disease. Other modalities include CT scans, which have proven to be more sensitive for detecting peripheral lesions [6]. Further diagnostic steps involve MRI, positron emission tomography (PET) scans, and biopsies of the lesion or lymph nodes. Current guidelines advise testing for advanced lung adenocarcinoma to identify key driver mutations [7]. CA-125 and CA-19-9 tumor markers may be elevated in small-cell lung cancer [8].

Management strategies depend on lymph node involvement and the extent of metastasis. In cases with localized solid nodules and minimal nodal involvement, surgical resection with lymph node biopsy is preferred. For patients who are not surgical candidates, radiotherapy with or without chemotherapy is recommended [1].

Given the diverse symptomatology of lung cancer, particularly when metastases are present, a misdiagnosis or delayed diagnosis is common. Conditions that can mimic lung cancer include respiratory diseases like tuberculosis and chronic obstructive pulmonary disease (COPD), as well as inflammatory conditions such as interstitial lung disease and sarcoidosis. Sarcoidosis, an immune-mediated granulomatous disease of unknown etiology, presents with systemic manifestations like fatigue, fever, weight loss, persistent dry cough, dyspnea, skin rashes, and blurred vision [9]. Further investigations, like chest X-rays, may typically reveal bilateral hilar and mediastinal lymphadenopathy, diffuse perilymphatic nodules, as well as interstitial fibrotic changes. Both the clinical picture as well as the supporting images are also possible presentations of adenocarcinoma, causing diagnosis uncertainty and further delay. There is a noticeable response to corticosteroids, making it the first line of treatment for this condition. It remains a diagnosis of exclusion, requiring both supportive clinical signs and pathological evidence for confirmation [10].

This report highlights a previously healthy, non-smoker, 49-year-old man whose symptoms and imaging results initially suggested sarcoidosis. Subsequent investigations, however, confirmed metastatic lung adenocarcinoma, highlighting how malignancy can closely imitate granulomatous disease both clinically and radiologically, causing uncertainty and delay in treatment.

## Case presentation

A previously healthy, 49-year-old Caucasian male developed a progressive, non-specific, multisystem illness over the course of 3 months.

During the first week of illness, the patient developed a flu-like syndrome accompanied by exertional dyspnea and a dry cough, which was more pronounced at night and disrupted his sleep. He subsequently reported intermittent left leg twitching at rest, lasting a few seconds and occurring in clusters of approximately 20 minutes, predominantly at night, and occasionally associated with tingling and numbness. He also described a sharp, burning pain radiating through the right shoulder and upper arm. In addition, he experienced gait imbalance with dizziness and nausea, particularly triggered by rapid movements. Additionally, he developed night sweats, insomnia, fatigue, reduced appetite, and visual auras. He experienced on-and-off generalized stabbing body aches, particularly of the cervical spine, long bones, hips, and ribs. Other symptoms included constipation without abdominal pain and increased fluid intake despite no change in urine output.

Symptoms were alleviated in the first two months with a combined inhaled corticosteroid, a long-acting beta-agonist, a leukotriene receptor antagonist, and an as-needed short-acting bronchodilator. After this period, the same symptoms recurred with a worsened intensity in addition to new-onset pleuritic left-sided chest pain when coughing.

The patient’s only regular medication includes a selective serotonin reuptake inhibitor to manage anxiety and depressive disorder. Otherwise, the patient was fit and active, with no chronic conditions. He is a lifelong non-smoker with no known exposure to occupational toxins.

On physical examination, the patient was afebrile, alert, and hemodynamically stable. Cardiopulmonary and abdominal examinations were unremarkable, with no organomegaly or ascites. No palpable cervical or axillary lymphadenopathy was detected. A neurological examination revealed mild unsteadiness during tandem gait but no focal motor deficits.

Given the patient’s non-specific multisystem symptoms, which progressively worsened over time, he was admitted for a comprehensive inpatient evaluation under internal medicine. At this stage, the patient's clinical features and initial adequate response to inhaled corticosteroids, long-acting beta-agonist, leukotriene receptor antagonist, and bronchodilator were highly suggestive of a granulomatous disease, and investigations included an extensive blood workup, a chest X-ray, and pulmonary function tests. In view of the concerning findings and the extent of his neurological symptoms, further advanced imaging with a brain MRI and a CT scan of the chest and abdomen was pursued. 

Laboratory investigations

Initial blood tests, including complete blood count and metabolic panel, were largely within normal limits (Table 1). Inflammatory markers were elevated, with increased CRP and ferritin, indicating systemic inflammation. Liver function tests demonstrated a mixed hepatocellular-cholestatic pattern, with a mild elevation in alanine aminotransferase (ALT), aspartate aminotransferase (AST), alkaline phosphatase (ALP), and bilirubin, suggesting possible hepatic involvement. Renal function, electrolytes, cardiac markers, and coagulation profile were normal. Immunological tests showed a mildly elevated immunoglobulin E (IgE) with a negative rheumatoid factor. These findings were non-specific but supported an inflammatory process.

**Table 1:** Laboratory values

Laboratory Marker	Patient Value	Reference Range
C-reactive protein	34.7 mg/L	<5 mg/L
Ferritin	454 µg/L	30–400 µg/L
Erythrocyte sedimentation rate	12 mm/hr	<20 mm/hr
Creatine kinase	48.7 U/L	22–198 U/L
Serum creatinine	83.5 µmol/L	60–110 µmol/L
Estimated glomerular filtration rate	99 mL/min/1.73 m²	>90 mL/min/1.73 m²
Glycated hemoglobin	5.0 %	<5.7 %
Total immunoglobulin E	117 kU/L	<100 kU/L
Rheumatoid factor	<10 IU/mL	<14 IU/mL
High-sensitivity cardiac troponin	4.55 ng/L	<14 ng/L
Total bilirubin	29.3 µmol/L	3–20 µmol/L
Direct bilirubin	10.4 µmol/L	<5 µmol/L
Alkaline phosphatase	418 U/L	40–130 U/L
Alanine aminotransferase	60.4 U/L	<41 U/L
Aspartate aminotransferase	36.1 U/L	<40 U/L
Serum albumin	37.8 g/L	35–50 g/L
Serum chloride	97.1 mmol/L	98–107 mmol/L

Pulmonary function tests

Spirometry was essentially normal, although mild small airway disease was detected with reduced mid-expiratory flow at 50% (MEF50). Fractional exhaled nitric oxide (FeNO) was within normal limits at 15 ppb.

Radiological imaging

**Figure 1:**
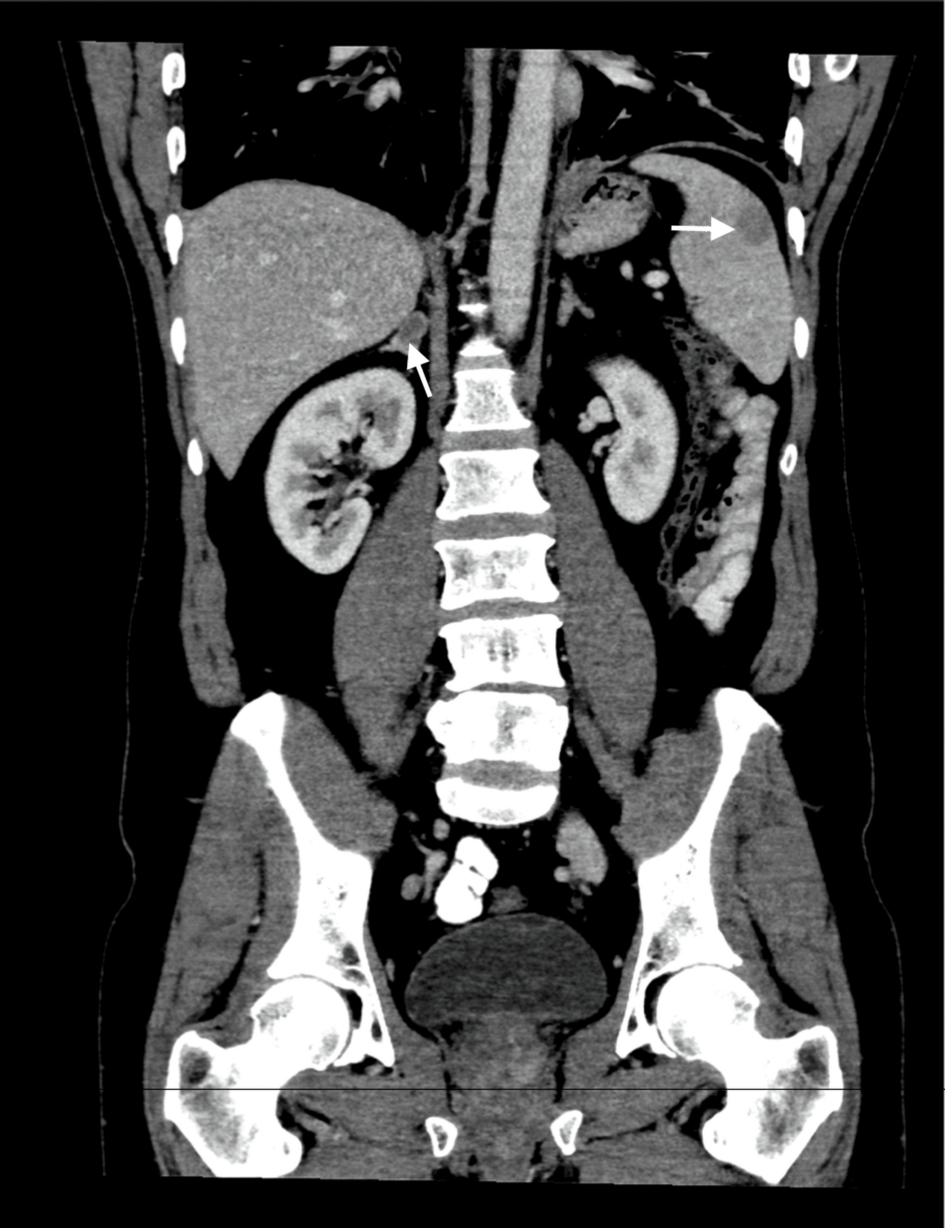
Contrast-enhanced CT scan of the abdomen and pelvis Solitary splenic and right adrenal nodules are visualized (arrows). These findings initially suggested sarcoidosis because the pattern of symmetrical lymphadenopathy and perilymphatic nodularity is typical of granulomatous disease. CT: computed tomography

A chest radiograph at presentation demonstrated coarse reticulation and interstitial thickening, consistent with interstitial lung disease. A CT scan of the chest, abdomen, and pelvis showed symmetrical hilar and mediastinal lymphadenopathy. There were diffuse reticulonodular opacities, perilymphatic nodular thickening, traction bronchiectasis, coalescing nodules, and fibrotic changes, accompanied by bilateral pleural effusion. Solitary splenic and right adrenal nodules were identified in the abdomen (Figure 1).

**Figure 2:**
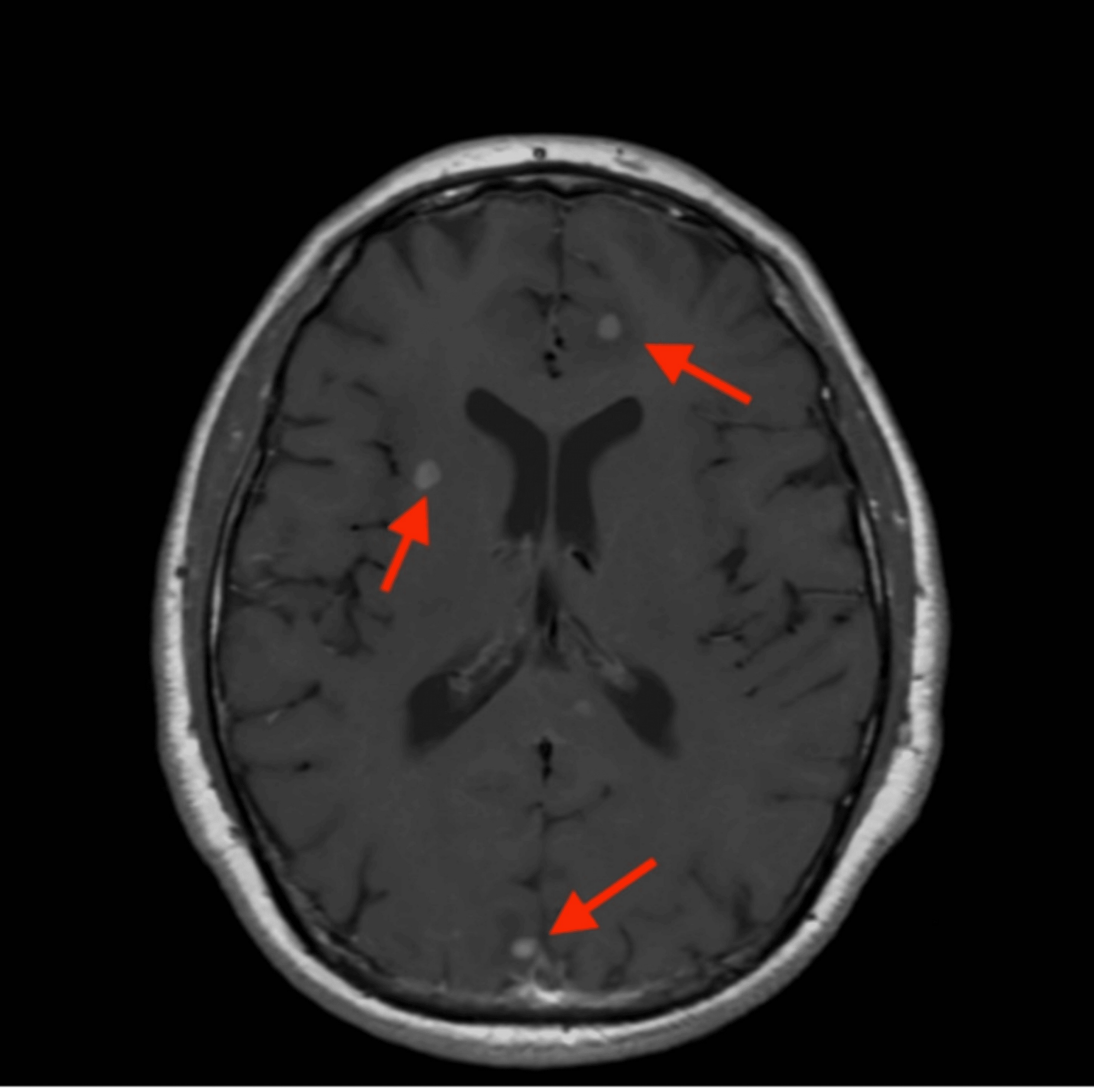
Brain MRI: axial FLAIR The image demonstrates multifocal enhancing lesions with surrounding edema, consistent with an inflammatory or granulomatous process. MRI: magnetic resonance imaging; FLAIR: fluid-attenuated inversion recovery

Brain MRI demonstrated multifocal enhancing lesions with surrounding edema (Figure 2).

**Figure 3:**
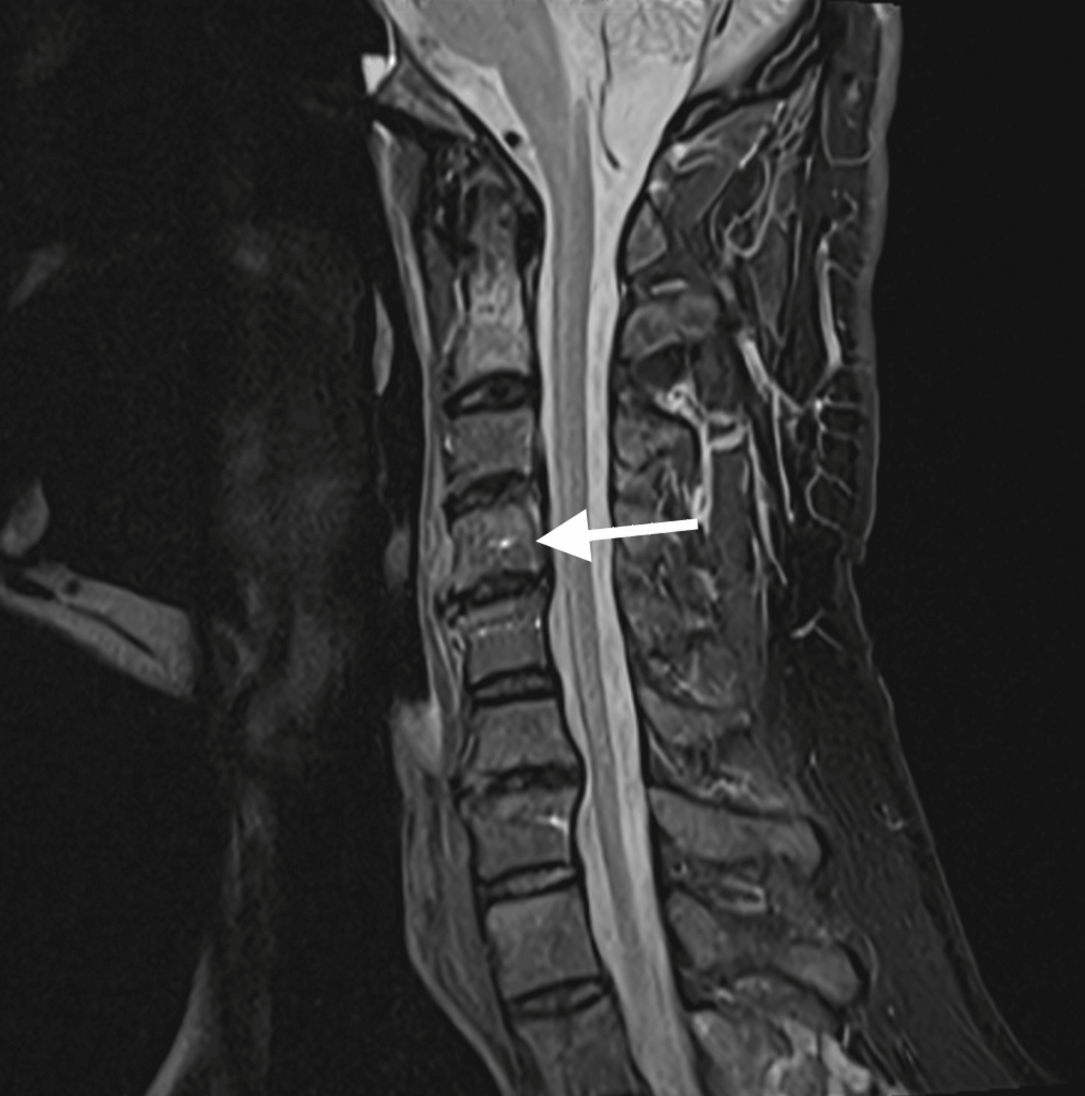
Spine MRI The image demonstrates patchy areas of abnormal marrow signal and contrast enhancement involving the cervical vertebrae, most prominently at the C4 vertebral body (arrow). MRI: magnetic resonance imaging

Spine MRI revealed patchy areas of abnormal bone marrow signal and contrast enhancement in C2, C4, and T7 with paraosseous soft tissue involvement (Figure 3).

The constellation of symmetrical hilar and mediastinal lymphadenopathy, perilymphatic nodular thickening, and reticulonodular opacities on CT, combined with normal calcium levels, elevated inflammatory markers, and an initially favorable response to corticosteroid therapy, raised clinical suspicion for sarcoidosis. These features aligned with a granulomatous inflammatory process and guided early diagnostic reasoning. Differential considerations at this stage included granulomatous or infective processes like sarcoidosis and metastatic disease.

**Figure 4:**
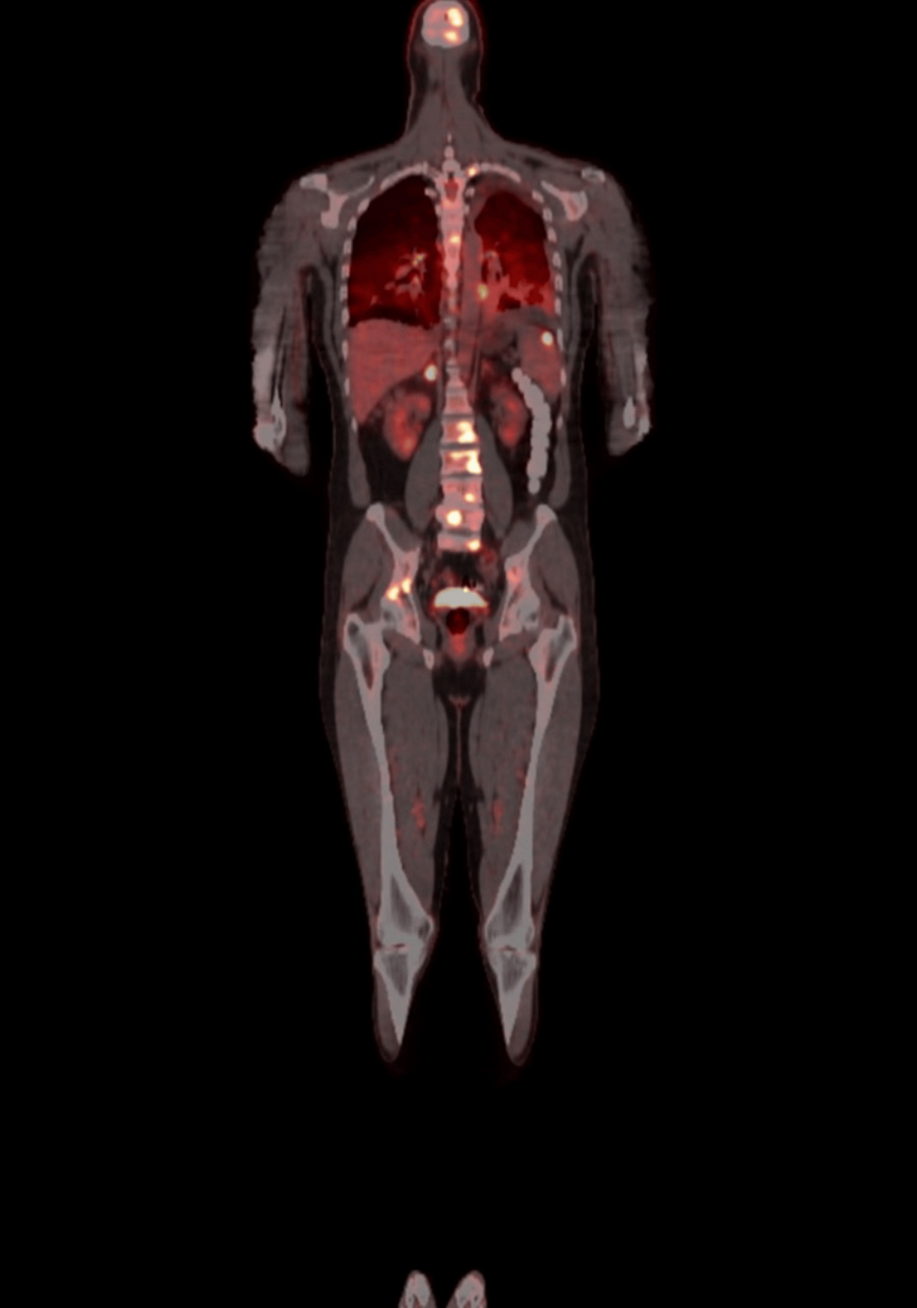
PET scan The scan demonstrates FDG-avid lesions involving the brain, mediastinal and hilar lymph nodes, lungs, spleen, right adrenal gland, liver, and multiple bones, consistent with widespread metastatic disease. PET: positron emission tomography; FDG: fluorodeoxyglucose

A PET scan was advised, which revealed an FDG-avid brain, lymph node, lung, spleen, right adrenal, and liver, as well as widespread bone lesions suggestive of metastasis (Figure 4).

Biopsy and final diagnosis

The key diagnostic turning point was the PET-CT, which demonstrated widespread FDG-avid lesions in the brain, lymph nodes, spleen, liver, adrenal gland, and bones, prompting reconsideration of sarcoidosis and raising suspicion for metastatic malignancy. A definitive diagnosis was established through a cervical lymph node biopsy, which confirmed metastatic adenocarcinoma consistent with a lung primary (Stage IV). Detailed histopathological descriptors and immunohistochemical results were not available in the final institutional pathology report.

Treatment and follow-up

Following diagnosis, the patient was referred for integrated oncological and palliative care. Molecular profiling identified a ROS1 rearrangement, and he was commenced on entrectinib monotherapy. The patient continues under regular oncologic follow-up with ongoing management aimed at disease control and symptom relief.

## Discussion

This case highlights the diagnostic complexity of a 49-year-old, previously healthy male who presented with nonspecific systemic and respiratory symptoms, initially suggestive of sarcoidosis. Clinical features, such as progressive dyspnea, dry cough, and constitutional symptoms, alongside imaging findings of bilateral hilar and mediastinal lymphadenopathy, supported the provisional diagnosis, especially given the initial corticosteroid responsiveness. However, the subsequent development of pleuritic chest pain and neurological symptoms, along with brain MRI findings of multifocal lesions, prompted a reconsideration of alternative diagnoses. PET-CT identified widespread FDG-avid lesions, with definitive diagnosis of metastatic lung adenocarcinoma being confirmed through biopsy.

This discussion focuses on reviewing the diagnostic workup and misleading findings that contributed to the delay in diagnosis, highlighting how the entire process exemplified anchoring bias [11]. The discussion will also review a similar case from the literature in which lung cancer was initially misdiagnosed.

One of the most misleading aspects of this case was the radiologic mimicry of sarcoidosis. Chest CT showed symmetrical bilateral hilar and mediastinal lymphadenopathy, perilymphatic nodules, reticulonodular opacities, fibrosis, traction bronchiectasis, and pleural effusions, features closely resembling stage IV sarcoidosis as per the Scadding Criteria [12]. The absence of a discrete central mass further reinforced a non-malignant impression. Laboratory findings were also inconclusive: angiotensin-converting enzyme (ACE), erythrocyte sedimentation rate (ESR), and rheumatoid factors were normal [13], while elevated CRP, ferritin, and IgE were nonspecific. Mild elevation of liver enzymes, which is seen in this case, is commonly seen in sarcoidosis. Tumor markers were not assessed at this stage, contributing to the delayed consideration of malignancy.

Neurological symptoms, including dizziness and visual auras, led to a brain MRI showing multifocal lesions with peripheral white matter edema; these non-specific findings could suggest a diagnosis of neurosarcoidosis, metastases, or infection. Given prior evidence favoring inflammation, the sarcoidosis diagnosis was further anchored. Partial symptom relief with corticosteroids and bronchodilators added a false sense of reassurance, despite these therapies being nonspecific and potentially masking malignancy.

PET-CT revealed FDG-avid lesions across multiple organs, raising suspicion for malignancy but not definitively ruling out sarcoidosis, which, to a lesser degree, can also show hypermetabolic uptake [14]. Ultimately, diagnosis hinged on histopathology: a cervical lymph node biopsy confirmed metastatic pulmonary adenocarcinoma. This case highlights the limitations of imaging and laboratory data in complex presentations and reinforces the essential role of tissue biopsy in achieving diagnostic certainty [15].

There is a notable lack of literature describing cases where lung cancer is initially misdiagnosed as sarcoidosis without evidence of true concurrent disease. Most published cases focus on the coexistence of sarcoidosis and lung cancer [16] or sarcoid-like reactions secondary to malignancy [17]. However, one case, described by Shin et al. [18], closely resembles this, detailing a patient with lung adenocarcinoma that was misdiagnosed as worsening pulmonary sarcoidosis due to initial biopsy findings of noncaseating granulomas. Similar to this case, the patient received corticosteroid therapy based on this presumptive diagnosis, which led to a delay in recognizing the underlying malignancy. Unlike this case, which left open the possibility of true sarcoidosis, our patient had no histopathological or clinical evidence of sarcoidosis after cervical lymph node biopsy confirmed primary lung cancer. This further emphasizes the diagnostic challenge in distinguishing between sarcoidosis, sarcoid-like reactions, and malignancy, especially when granulomatous inflammation is present, and highlights the importance of re-evaluating non-responders to corticosteroid therapy to avoid delays in cancer diagnosis.

Lung cancer, once considered a disease predominantly affecting older adults with a smoking history, is increasingly being diagnosed in younger individuals, including non-smokers. Studies have shown that approximately 10% of lung cancer cases now occur in patients under the age of 55, with around 1% diagnosed before the age of 45 [19]. In a Peruvian cohort, 4.3% of lung cancer patients were aged 40 or younger, most of whom were female non-smokers presenting with advanced adenocarcinoma and poor survival outcomes (median OS: 8.2 months) [20]. Similar trends were seen in northern China, where younger patients had a higher proportion of EGFR mutations, a family history of cancer, and late-stage presentation. A Turkish study found that the median age at lung cancer diagnosis was 62, 9 years younger than in the U.S., highlighting regional variability in onset patterns [21]. Furthermore, in the United States, lung cancer incidence in women under 55 has equaled or surpassed that of men in many states, despite lower smoking rates among women, suggesting other etiologic factors at play such as environmental exposures or genetic susceptibility [22]. These demographic and molecular shifts identify the importance of early consideration of lung cancer in younger patients, even in the absence of traditional risk factors.

Given the evolving nature of this patient's presentation, lung cancer could have been reasonably considered earlier in the diagnostic process, particularly at the point when neurological symptoms began to emerge alongside systemic signs of disease progression. The presence of visual auras, leg tingling, and gait imbalance prompted a brain MRI, which resulted in findings highly atypical for sarcoidosis alone and should have raised a strong suspicion for metastatic disease, particularly in the absence of other convincing signs of neurosarcoidosis. Additionally, the appearance of pleuritic chest pain, progressive systemic symptoms, such as significant fatigue, weight loss, and generalized body aches, combined with only partial and temporary response to corticosteroid therapy, should have triggered a reassessment of the working diagnosis. At this juncture, incorporating malignancy into the differential and expediting a tissue biopsy could have potentially led to an earlier diagnosis and initiation of appropriate oncologic management.

Fortunately, in recent years, significant progress has been made in the early detection and diagnostic accuracy of lung cancer, particularly NSCLC. One of the most impactful developments has been the use of low-dose computed tomography (LDCT) for screening high-risk populations. The landmark National Lung Screening Trial (NLST) demonstrated a 20% reduction in lung cancer mortality among individuals screened with LDCT compared to those screened with chest radiographs, establishing LDCT as the standard for early detection in eligible patients [23]. Similarly, the NELSON trial (Nederlands-Leuvens Longkanker ScreeningsONderzoek/Dutch-Belgian Randomized Lung Cancer Screening Trial) confirmed this benefit in a European population, further solidifying the role of LDCT in clinical practice [24].

Despite these advancements, our patient would not have qualified for routine lung cancer screening under current guidelines, as he did not meet the criteria for high-risk individuals. Most screening protocols, including those recommended by the U.S. Preventive Services Task Force (USPSTF), are designed for adults aged 50-80 years with a 20-pack-year smoking history who currently smoke or have quit within the past 15 years.

Our patient, a 49-year-old lifelong non-smoker with no known occupational exposures or family history of lung cancer, fell outside these parameters. This highlights a significant limitation of the existing screening criteria, which may miss early or atypical presentations of lung cancer in younger, low-risk individuals, particularly those with non-specific symptoms and no conventional risk factors. At diagnosis, his disease was already advanced and metastatic, precluding curative options.

## Conclusions

In conclusion, this case underscores the diagnostic complexity that arises when malignancy clinically and radiographically mimics granulomatous diseases such as sarcoidosis. Initial improvement with corticosteroid therapy and imaging findings consistent with sarcoidosis delayed the recognition of metastatic lung adenocarcinoma. This highlights the importance of maintaining a broad differential diagnosis, particularly when clinical features are atypical or the treatment response is incomplete.

The case also emphasizes the limitations of imaging in distinguishing granulomatous from malignant lymphadenopathy and the critical role of early tissue sampling in ambiguous presentations. Above all, it illustrates how cognitive biases, such as anchoring bias, can delay accurate diagnosis and impact outcomes. Clinicians should maintain a high index of suspicion for malignancy in sarcoid-like presentations, especially when corticosteroid response is partial or symptoms progress.
